# Proteomic profiling of cellulase-aid-extracted membrane proteins for functional identification of cellulose synthase complexes and their potential associated- components in cotton fibers

**DOI:** 10.1038/srep26356

**Published:** 2016-05-19

**Authors:** Ao Li, Ruyi Wang, Xianliang Li, Mingyong Liu, Jian Fan, Kai Guo, Bing Luo, Tingting Chen, Shengqiu Feng, Yanting Wang, Bingrui Wang, Liangcai Peng, Tao Xia

**Affiliations:** 1 National Key Laboratory of Crop Genetic Improvement and National Centre of Plant Gene Research, Huazhong Agricultural University, Wuhan 430070, China; 2 Biomass and Bioenergy Research Centre, Huazhong Agricultural University, Wuhan 430070, China; 3 College of Plant Science and Technology, Huazhong Agricultural University, Wuhan 430070, China; 4 College of Life Science and Technology, Huazhong Agricultural University, Wuhan 430070, China; 5 School of Biology and Food Engineering, Changshu Institute of Technology, Changshu 215500, China

## Abstract

Cotton fibers are an excellent model for understanding of cellulose biosynthesis in higher plants. In this study, we determined a high cellulose biosynthesis activity *in vitro* by optimizing biochemical reaction conditions in cotton fibers. By adding a commercial cellulase enzyme into fibers extraction process, we extracted markedly higher levels of GhCESA1 and GhCESA8 proteins and observed an increase in β-1,4-glucan and β-1,3-glucan products *in vitro*. LC-MS/MS analysis of anti-GhCESA8-immunoprecipitated proteins showed that 19 proteins could be found in three independent experiments including four CESAs (GhCESA1,2,7,8), five well-known non-CESA proteins, one callose synthase (CALS) and nine novel proteins. Notably, upon the cellulase treatment, four CESAs, one CALS and four novel proteins were measured at relatively higher levels by calculating total peptide counts and distinct peptide numbers, indicating that the cellulase-aid-extracted proteins most likely contribute to the increase in β-glucan products *in vitro*. These results suggest that the cellulase treatment may aid to release active cellulose synthases complexes from growing glucan chains and make them more amenable to extraction. To our knowledge, it is the first time report about the functional identification of the potential proteins that were associated with plant cellulose and callose synthases complexes by using the cellulase-aided protein extraction.

Cotton fibers are single epidermal cells that contain more than 90% cellulose at the mature stage, which have been regarded as ideal materials for analysis of plant cellulose biosynthesis^1^,^2^. In higher plants, cellulose is synthesized at the plasma membrane by a large rosette complex that is believed to simultaneously synthesize the β-1,4-glucan chains forming microfibrils^3^,^4^,^5^. Although the cellulose synthase complex plays the main role in microfibril formation and assembly, much remains unknown about its associated-components^6^,^7^. In *Arabidopsis*, a number of cellulose synthase subunits (CESAs) are required for cellulose synthesis in primary cell walls^8^,^9^,^10^,^11^,^12^,^13^, whereas three nonredundant CESAs are essential for cellulose synthesis in secondary cell walls^14^,^15^,^16^. The CESA isoforms found in both primary and secondary cell walls have been shown to interact with one another through co-immunoprecipitation assays^12^,^13^,^16^. More recently, it has been reported that four GhCESA isoforms (GhCESA1, 2, 7, 8) are involved in cellulose biosynthesis of secondary cell wall and GhCESA3, 5, 6, 9, 10 are associated with primary cell wall formation in cotton^2^,^17^,^18^,^19^.

Several non-CESA proteins have been reported to be involved in cellulose biosynthesis, orientation and assembly in plants, such as sucrose synthase (SUSY), Korrigan (KOR), actin and tubulin proteins^20^,^21^,^22^,^23^,^24^,^25^,^26^,^27^,^28^,^29^,^30^,^31^,^32^,^33^. However, there is currently little information on whether non-CESA proteins are associated with the CESA complex. To answer this question, it is essential to isolate an intact, pure, functional CESA complex. Using blue native polyacrylamide gel electrophoresis (BN-PAGE) of solubilized cell extracts, Wang *et al*.^34^ have demonstrated the presence of primary cell wall CESAs as components of a large (840 kDa) complex, although the composition of this complex remains to be determined. In addition, Atanassov *et al*.^35^ employed an epitope tagging approach to purify a secondary cell wall CESA-containing complex under nondenaturing conditions. The harvested CESA complex consisted solely of the three known secondary cell wall CESAs. In *Populus* xylem tissues, five typical secondary cell wall CESAs can be detected in the pulled down by an anti-PtiCESA7-A antibody^36^. A number of non-CESA proteins have also been found through immunoprecipitation, but little is known about their associations with the CESA complex.

As CESA proteins are quite unstable, cellulose synthase activity has not been well characterized *in vitro*^37^,^38^,^39^. In addition, large quantities of β-1,3-glucan and small amounts of β-1,4-glucan are synthesized *in vitro* when incubating detergent membrane extracts with UDP-glucose^37^,^40^. Therefore, the instability of CESA complex with large amounts of β-1,3-glucan products leads to technical difficulties in detecting high activity of cellulose biosynthesis *in vitro*.

It has been proposed that wound-induced callose is produced by cellulose synthase as a result of a loss of accessory proteins required for cellulose biosynthesis or a change of conformation during protein extraction^41^. However, this hypothesis is under debate because a plant callose synthase (designated CALS) has been identified that does not show any significant similarity to plant CESAs^42^,^43^. In particular, CALS does not contain the D, D, D, QXXRW motif^44^, and its molecular weight is in the range of 190–220 kDa, compared with 110–120 kDa for CESAs^45^,^46^. Thus, whether cellulose synthase can catalyze the formation of callose *in vitro* remains unclear until a fully functional cellulose complex is isolated^35^,^40^ or an active cellulose synthase preparation devoid of callose synthase activity becomes available^47^. In addition, it is important to detect the callose synthase complex in plants.

As cotton fibers are excellent experimental materials for cellulose biosynthesis, we obtained high cellulose and callose biosynthesis activities *in vitro* by adding commercial cellulase during the cotton fiber extraction process in this work. Then, we could demonstrate a powerful approach for functional identification of the components that are associated with cellulose and callose synthases complexes by performing a proteomic profiling of the cellulase-aid-extracted proteins in cotton fibers.

## Results

### Detection of β-1,4-glucan and β-1,3-glucan products *in vitro*

**Figure 1:**
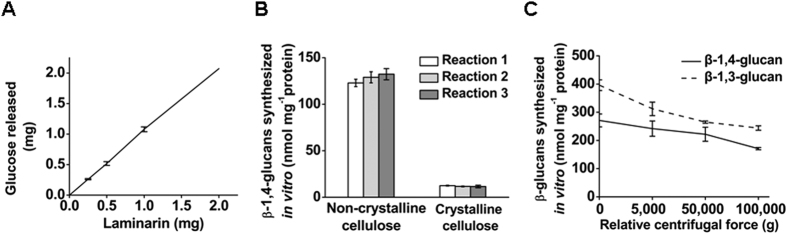
Detection of β-glucan products *in vitro.* (**A**) Laminaria hydrolysis using a mixture of endo-β-1,3-glucanase and exo-β-1,3-glucanase and the quantity of released glucose determined via the anthrone-H_2_SO_4_ method and verified via GC/MS; error bars indicate SD (*n* = 3). (**B**) β-1,4-Glucan products synthesized *in vitro* after endo-β-1,3-glucanase and exo-β-1,3-glucanase hydrolysis to remove callose: the total glucose released by the β-1,4-glucanases was defined as non-crystalline cellulose, and the remaining pellet was defined as crystalline cellulose; All experiments were conducted in biological triplicate and data are presented as the mean ± SD (*n* = 3). (**C**) β-1,4-Glucan and β-1,3-glucan products synthesized using different centrifugation speeds for the isolation of detergent-soluble membrane proteins; error bars indicate SD (*n* = 3).

To examine the specificity *in vitro* of the commercial β-1,3-glucanase and β-1,4-glucanase activities, we used laminaria and carboxymethyl cellulose sodium (CMC) as substrates, respectively, and the released glucose and oligosaccharides were detected via the anthrone-H_2_SO_4_ method and further confirmed through GC/MS. We found that β-1,3-glucanase could completely hydrolyze laminaria (Fig. 1A) but showed no activity in relation to CMC (data not shown). In contrast, β-1,4-glucanase exhibited strong activity with CMC (data not shown) but did not release any product from laminaria. To detect β-1,3-glucan and β-1,4-glucan products *in vitro*, we initially performed β-1,3-glucanase digestion to completely remove the synthesized callose and then defined the remaining pellet as the cellulose (total β-1,4-glucan) product. Furthermore, we found that approximately 90% of the total β-1,4-glucan product could be effectively digested by β-1,4-glucanases, and the remaining pellet was defined as crystalline cellulose. In this study, we observed that less than 10% of the total β-1,4-glucan product was crystalline cellulose (verified by linkage analysis with GC/MS), though this proportion was slightly altered by different reactions (Fig. 1B). Hence, we only present the data for the total β-1,4-glucan products in this report.

To assay glucan biosynthesis activity *in vitro*, we tested different ultracentrifugation methods for isolating total crude membrane proteins from cotton fibers. First, we compared sucrose density and sedimentation centrifugation at 100000 *g*. The results showed that the quantity of β-1,4-glucan and β-1,3-glucan products synthesized *in vitro* using membrane proteins extracted via the sucrose density method was 2-fold lower compared with those extracted via sedimentation velocity centrifugation (*t*-test, *P* < 0.01, *n* = 3). However, the ratios of β-1,4-glucan to β-1,3-glucan obtained using the two methods were similar (Table 1). Thus, the sedimentation velocity centrifugation method was used for total crude membrane extraction in this work. Consequentially, we performed centrifugation at different speeds to precipitate the digitonin-insoluble membrane proteins from cotton fibers and did comparisons with the observed digitonin-soluble membrane synthase activity *in vitro*. The data indicated that the quantities of both β-1,4-glucan and β-1,3-glucan products were decreased as centrifugation speeds were increased (Fig. 1C). Hence, total crude membrane extraction was performed by centrifuging homogenate at 5,000 *g* in this work.

### Extraction of cellulose synthase enzymes through cellulase treatment

**Figure 2:**
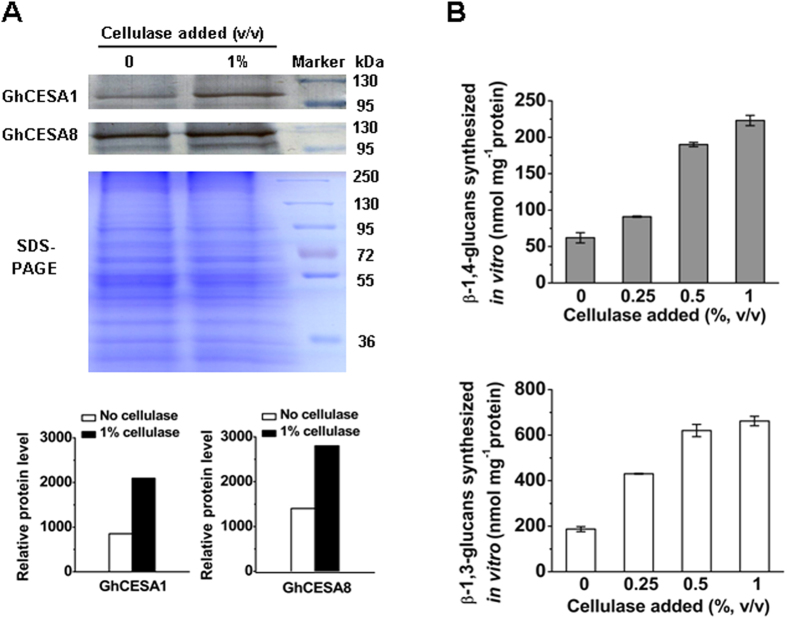
Identification of GhCESA1 and GhCESA8 proteins and detection of β-1,4-glucan and β-1,3-glucan products *in vitro* from total membrane extracts of cotton fibers treated with/without cellulase. (**A**) Western blot analysis of GhCESA1 and GhCESA8 (upper lane), separation of total membrane proteins by SDS-PAGE and staining with Coomassie Blue (middle lane) and relative levels of both proteins (lower lane). The relative protein level was measured by calculating the protein band area x protein band intensity. (**B**) Products of β-1,4-glucans (upper lane) and β-1,3-glucans (lower lane) synthesized *in vitro* from cotton fiber extracts treated with different concentrations of cellulases; the data are presented as the mean ± SD (*n* = 3).

To effectively extract cellulose synthase proteins, we attempted to add commercial cellulase to the extraction buffer for cotton fibers. As a result, much greater quantities of GhCESA1 and GhCESA8 proteins were detected in the membrane extract with the cellulase treatment compared with the control (no cellulase) (Fig. 2A). Accordingly, the yields of both β-1,4-glucans and β-1,3-glucans synthesized *in vitro* were dramatically increased through cellulase treatment (Fig. 2B). The quantity of β-1,4-glucan was increased by 1.5–3.6 fold (*t*-test, *P* < 0.01 or 0.05, *n* = 3), while that of β-1,3-glucan was increased by 2.3–3.5 fold (*t*-test, *P* < 0.01, *n* = 3) as the added cellulase was increased from 0.25% to 1% (Supplementary Table S1). However, the ratios of β-1,4-glucan to β-1,3-glucan remained similar in the treatments involving different concentrations of cellulase. The data indicated that cellulase treatment may lead to effective extraction of cellulose and callose synthases in cotton fibers to obtain high yields of glucans synthesized *in vitro*.

### Immunoprecipitation of the cellulose synthase complex

**Figure 3:**
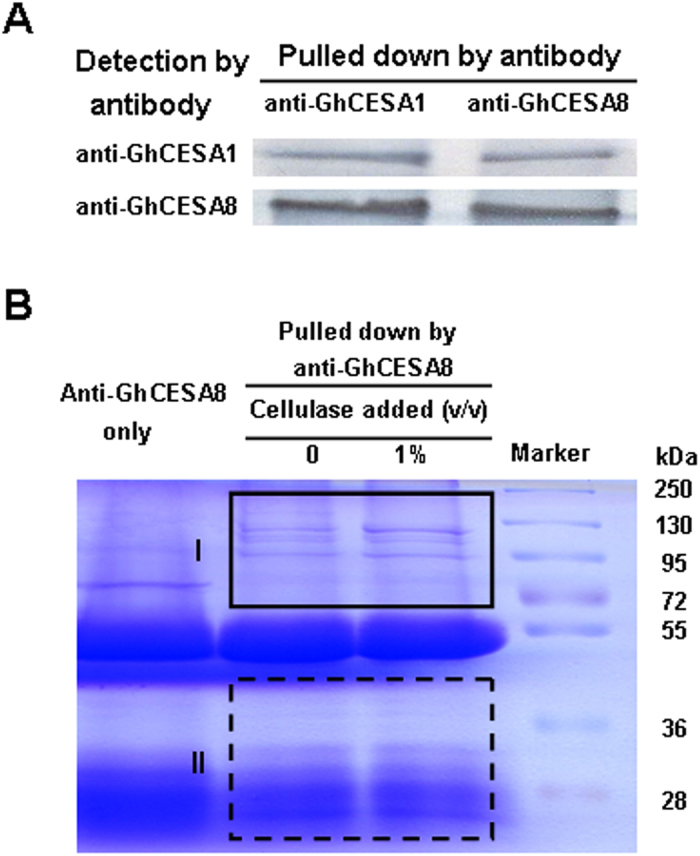
Immunoprecipitation of the putative cellulose synthase complexes with anti-GhCESA1 and anti-GhCESA8. (**A**) Western blot analysis of GhCESA1 and GhCESA8 in the cellulose synthase complex precipitated with anti-GhCESA1 and anti-GhCESA8. (**B**) Separation of the precipitated proteins via SDS-PAGE and staining with Coomassie blue; the frames indicate the sections of the gels excised for LC-MS/MS analysis.

As GhCESA1 and GhCESA8 have been identified as the major enzymes involved in cellulose biosynthesis in cotton fibers (Supplementary Table S2)^2^,^17^,^18^,^19^, we used antibodies against these proteins to pull down the CESA complex through co-immunoprecipitation (Fig. 3A; Supplementary Fig. S1). Western blot analysis indicated that both GhCESA1 and GhCESA8 were precipitated with each antibody, suggesting that the antibodies can be applied to pull down the CESA complex from cotton fibers. As GhCESA8 is an enhancer specific for massive cellulose production in cotton fibers^2^, we chose to use anti-GhCESA8 for the following co-immunoprecipitation experiments (Fig. 3B). Upon SDS-PAGE separation, several protein bands ranging from 90 to 130 kDa in size were observed following anti-GhCESA8 precipitation from cotton fiber extracts, which were not observed in the control (anti-GhCESA8 only). Furthermore, when commercial cellulase (1%) was added to the extraction buffer for cotton fibers, the obtained protein bands were markedly more intense than in the control (no cellulase). This result suggested that the CESAs of cotton fibers could be effectively extracted using the described cellulase treatment.

### Analysis of the CESA complex following anti-GhCESA8 precipitation

As described above, the SDS-PAGE gels were cut into two sections for the identification of all potential CESA complex, and the un-cut portion of the gel was removed, being considered to contain anti-GhCESA8 compounds (Fig. 3B). To identify the GhCESA complex, we performed a proteomic analysis of the precipitated proteins from the mature cotton fibers using the LC-MS/MS method, and we observed a large number of peptide sequences corresponding to CESAs in three independent experiments (Table 2). Although GhCESA1, 2, 7 and 8 could be detected without cellulase treatment (Table 2; Supplementary Table S3), much greater quantities of either total peptide counts or distinct peptide numbers for the four CESAs were found in the presence of cellulase, consistent with the observations of increased GhCESA1 and 8 protein levels by cellulase treatment (Figs 2 and 3). As total peptide counts and distinct peptide numbers have been applied for protein relative level measurement^48^,^49^, we used both peptide parameters to find out all proteins that can be enhanced by cellulase treatments in three independent experiments.

In addition, because of the high identity (94.7%) of GhCESA7 and GhCESA8 at the amino acid sequence level, specific peptides of these two CESAs were identified in much smaller quantities compared with GhCESA1 and 2 (Supplementary Table S3). On the other hand, the data supported that four GhCESA isoforms (GhCESA1, 2, 7 and 8) are involved in secondary cell wall biosynthesis in cotton fibers^2^.

### Identification of all proteins following anti-GhCESA8 precipitation

Upon co-immunoprecipitation with anti-GhCESA8, we identified 32 proteins in three independent experiments. To identify the proteins that have specific associations with CESA complex, LC-MS/MS analysis of immunoprecipitation was conducted using anti-GhCESA8 and the pre-immune serum that was defined as control for excluding non-specific proteins (Supplementary Table S4). Among the identified 32 proteins, 11 proteins were specifically found in the precipitation using anti-GhCESA8, other than using the pre-immune serum. Those 11 proteins included callose synthase (CALS), sucrose synthase, beta-tubulins and others (Supplementary Table S4, II). In addition, another eight proteins exhibited total peptide counts of anti-GhCESA8 by 2–4 folds more than that of the pre-immune serum, including four CESAs, endo-1,4-beta-glucanase (KOR) and others (Supplementary Table S4, I). It suggests that those identified 19 proteins should have strong and specific associations. By comparison, the remaining 13 proteins were considered with possible non-specific associations, as the total peptide counts using anti-GhCESA8 were less than 2-folds compared with the pre-immune serum (Supplementary Table S4, III).

**Figure 4:**
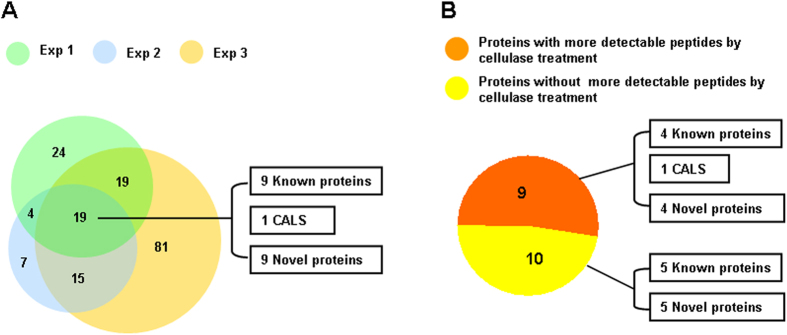
Numbers of detectable proteins observed in LC-MS/MS analysis following immunoprecipitation with anti-GhCESA8. (**A**) Numbers of anti-GhCESA8-precipitated proteins obtained in three independent experiments. (**B**) Proteins showing more total peptide counts and distinct peptide numbers (orange) or without more detectable ones (yellow) upon cellulase treatment.

Taken together, the identified 19 specific proteins included nine well-known proteins (four CESAs and five non-CESAs), one CALS and nine novel proteins (Fig. 4A; Supplementary Table S5, Supplementary dataset 1). Among the five non-CESA proteins (Table 3), sucrose synthase and endo-1,4-β-glucanase (KOR) have been characterized to strongly involve in cellulose biosynthesis, and three tubulin isoforms are associated with cellulose orientation and assembly^20^,^21^,^22^,^23^,^24^,^25^,^26^,^27^,^28^,^29^,^30^,^31^,^32^,^33^. Therefore, this study could support the notion that these five non-CESA proteins were associated with the CESA complex due to their co-immunoprecipitation with anti-GhCESA8.

Based on the cellulase treatment of cotton fibers, the identified 19 proteins could be further classified into two categories (Fig. 4B). Notably, nine proteins presented much higher levels of total peptide counts and distinct peptide numbers in the LC-MS/MS analysis following cellulase treatment, including four CESA proteins, as described above (Table 2), one CALS and other four proteins. In particular, the cellulase enhancement of CALS was consistent with the observed increase in the quantity of the β-1,3-glucan product *in vitro* due to cellulase treatment. This result also confirmed that CALS is likely to be closely associated with the CESA complex, based upon their effective co-extraction following cellulase treatment and co-precipitation using anti-GhCesA8. As a comparison, the remaining ten proteins were not found with the increased total peptide counts (or distinct peptide numbers) from the cellulase treatment (Fig. 4B; Table 3).

### Classification of the CESA and CALS-associated proteins

Using a public database, we searched for the *Arabidopsis* homologs of the 19 identified cotton fiber proteins and observed the co-expression patterns of the homologous genes in 64 tissues covering almost all periods of the *Arabidopsis* life cycle (Supplementary Table S6). Based on the hierarchical clustering analysis, the co-expression patterns could be typically classified into two groups (Supplementary Fig. S2). Group I covered three secondary cell wall *AtCESAs* and seven non-CESA genes, group II contained *AtCALS9* and six other genes.

Gene co-expression analysis has been extensively applied to predict potential protein interactions or associations^50^,^51^. According to the obtained microarray data, we performed a correlation analysis of the expression patterns of the homologous *Arabidopsis* genes. The *Arabidopsis* homologs of the five well-known cotton non-CESA genes having possible association with CESA complex (Table 3) displayed significant co-expression, at the *P* < 0.01 and 0.05 levels, with the secondary cell wall *AtCESAs* (Supplementary Table S7), indicating that the applied correlation analysis should be powerful for detecting CESA-associated components. Furthermore, another six non-CESA genes also exhibited a significantly positive correlation with the secondary cell wall *AtCESAs*, and at least two genes were found to show more detectable total peptide counts (or distinct peptide numbers) in cotton fibers upon cellulase treatment (Table 4; Supplementary Table S7). In comparison, three *Arabidopsis* gene homologs displayed a significantly positive correlation with the callose synthase gene *AtCALS9*, and two genes showed more detectable total peptide counts (or distinct peptide numbers) in cotton fibers upon cellulase treatment (Table 4; Supplementary Table S7). Furthermore, we compared the anti-CESA- immunoprecipatated components between cotton fibers and *Populus* xylem fibers^36^, and 12 proteins were found in both plant species, including four GhCESAs, five well-known non-CESA proteins, one GhCALS and two novel proteins (Table 5).

## Discussion

### Effect of different centrifugation methods on β-glucan products *in vitro*

Various centrifugation methods have been extensively applied for the separation and isolation of biological compounds^52^,^53^,^54^. In this study, we compared two distinct methods for the extraction of total membrane-associated proteins: sucrose density centrifugation and sedimentation velocity centrifugation. The sucrose density method resulted in relatively low yields of β-1,4-glucan and β-1,3-glucan products, possibly due to the technique difficulty of avoiding the collection of cytoplasmic compounds, including proteinases and other inhibitory factors, from the buffer-sucrose interface following centrifugation. With respect to the decreased quantities of glucan products (per mg of protein *in vitro*) obtained under the increasing centrifugation speeds used for the removal of digitonin-insoluble membrane proteins (Fig. 1C), we assumed that the intact cellulose synthase complex may be partially lost in the reaction, due to a loss of large-size membrane pellets under high-speed centrifugation.

### Effective extraction of the cellulose synthase complex upon the addition of cellulose

To enhance the quantities of β-1,4-glucan products obtained *in vitro*, we attempted to efficiently extract the intact cellulose synthase complex by treating cotton fibers with commercial cellulase. Accordingly, the cellulase treatment strongly enhanced either the β-glucan products measured *in vitro* or the two major CESA isoforms (GhCESA1 and GhCESA8). As the termini of β-1,4-glucans have been observed to associate with cellulose synthase complex in the plasma membrane^6^,^55^,^56^, the applied cellulase treatment may have played a role in effective CESA disassociation from the β-1,4-glucans. Although the plant cellulase genes (*KOR*/*AtRsw2*) have been reported to involve in cellulose biosynthesis^21^,^22^,^24^,^32^,^33^,^38^,^57^, we did not find that the β-glucan products obtained *in vitro* could be increased by adding various concentrations of commercial cellulases to the reaction buffer (data not shown), suggesting that commercial cellulases do not participate in β-glucan biosynthesis. This finding is consistent with the recent report that only plant specific family of *GH* genes (*Kor*) is involved in cellulose biosynthesis^32^,^33^,^57^.

### Functional identification of potential CESA-associated components

Genetic mutants and biochemical co-immunoprecipitation analyses have been applied to characterize three CESA isoforms as the essential cellulose synthase complex typical of secondary cell wall biosynthesis in *Arabidopsis* and other plants^14^,^15^,^16^,^51^,^58^,^59^,^60^. However, recent reports have indicated that one and two more isoforms are required in cotton fibers and *Populus* xylem fibers, respectively, which are extremely rich in cellulose contents^2^,^36^. These findings are supported by the observation in the present study that using cellulase-aid-extracted CESAs results in increased β-1,4-glucan products *in vitro*. The four well-known GhCESAs and two novel proteins are thus proposed as a major contribution to the high β-1,4-glucan biosynthesis detected *in vitro*, due to their remarkable enhancement through cellulase treatment. Notably, among the two novel proteins (Table 4), the vacuolar H^+^ -ATPase catalytic subunit has been reported to regulate the CESA complex trafficking to the plasma membrane^61^. However, while the four GhCESA isoforms and other novel proteins require genetic and biochemical tests to determine their involvement in cellulose biosynthesis, to our knowledge, this study at first time has demonstrated an applicable and powerful approach for functional identification of the potential CESA-associated components in plants.

Although neither well-known non-CESA proteins (SUSY, KOR and tubulins) nor four novel proteins showed any enhancement upon cellulase treatment, these proteins are expected to be associated with CESAs due to their co-extraction via anti-GhCESA8 immunoprecipitation in three independent experiments (Tables 3 and 4). As the five non-CESA proteins (SUSY, KOR and tubulins) have been well characterized in plants in relation to *in vivo* cellulose biosynthesis^20^,^21^,^22^,^23^,^24^,^25^,^26^,^27^,^28^,^29^,^30^,^31^,^32^,^33^, the four novel proteins have also been suggested to play roles in cellulose biosynthesis and assembly. In fact, the FLA (fasciclin-like arabinogalactan protein) proteins have been reported to have effects on cellulose biosynthesis^62^. However, despite that three independent experiments were performed for identifications of the anti-GhCESA8 immunoprecipitated proteins in this study, all of the novel proteins require further genetic analysis through overexpression or RNAi silencing of their encoding genes in cotton and/or other plants in the future.

On the other hand, SUSY and KOR are presumed to produce UDP-glucose and sterol glycoside for cellulose biosynthesis^38^,^63^, but both of the produced chemical compounds were provided at substantial levels *in vitro* in the reaction. Therefore, it is rational to infer that SUSY and KOR are not essential for enhanced β-1,4-glucan production *in vitro*. With respect to the SUSY and KOR showing non-enhancement from the cellulase-aid-extraction, we presume that both proteins may indirectly or weakly interact with CESA complexes. In addition, as tubulins are reported to regulate the *in vivo* orientation and deposition of β-1,4-glucans in plants^26^,^27^,^28^,^29^,^30^, these proteins may not be involved in cellulose biosynthesis *in vitro*, thus leading to the small proportion of crystalline β-1,4-glucan products that were not enhanced by the cellulase treatment applied in this study.

In addition, as the mature cotton fibers used in this study are typical secondary cell walls, we did not find any CESA proteins of primary cell walls in the cellulose synthase complex, such as the reported GhCESA3, 5, 6, 9, 10^2^,^17^,^18^,^19^. This may also explain that we could not detect the other protein that has been reported to involve in cellulose biosynthesis and assembly of primary cell walls in *Arabidopsis*, such as POM2/CSI1 protein^64^,^65^,^66^,^67^.

### Potential association between cellulose and callose synthase complexes

It has been reported that cellulose and callose can be synthesized *in vitro* by the same membrane-bound enzyme complexes, which use UDP-glucose as a sugar donor^37^,^38^. In this study, callose synthase was demonstrated to show a close association with the cellulose synthase complex, based on its effective precipitation with anti-GhCESA8 upon the cellulase treatment of cotton fibers. The fact that callose synthase is also found in CESA-precipitated proteins in *Populus* xylem fibers^36^ suggests that this enzyme should show an association with cellulose synthase complex in higher plants. Furthermore, the results of this study indicate that the callose synthase may interact with three other novel proteins for high callose production in cotton fibers (Table 4).

## Conclusion

Proteomics profiling anti-GhCESA8 immunoprecipitation products has revealed a total of 19 proteins, including including four CESAs (GhCESA1, 2, 7, 8), five well-known non-CESA proteins, one callose synthase (CALS) and nine novel proteins in cotton fibers. Based on the cellulase-aid-extraction of cotton fiber proteins and bioinformatics-analysis of *Arabidopsis* homolog, a hypothesis model could be proposed for functional characterization of cellulose and callose synthase complex and their potential associated-components in cotton fibers.

## Methods

### Plant materials

Upland cotton (*Gossypium hirsutum*) Huamian 99, provided by Dr. Guozheng Yang, was used in the experiments. The plants were grown in the experimental field of Huazhong Agricultural University. On the day of anthesis, flowers were tagged, and the corresponding bolls were harvested at 9, 14, 19 and 24 DPA (day past anthesis). After harvesting, the fibers were gently removed from the ovules, then quickly frozen in liquid nitrogen and stored at −80 °C until in use.

### Extraction of membrane proteins

Membrane proteins were extracted from cotton fibers using a modified procedure as described by Peng *et al*.^38^. Cotton fibers were removed from seeds at 24 DPA (extremely rich secondary cell walls) and ground to a fine powder in liquid nitrogen, and 7.0 g of this powder was extracted with 40 ml of ice-cold extraction buffer (50 mM Mops/NaOH buffer, pH 7.5, 0.25 M sucrose) containing protease inhibitors (1.0 mM PMSF, 1.0 μM pepstatin A, and 1.0 μM leupeptin). Then, the extracts were transferred to 15 ml tubes and centrifuged at 2,000 *g* for 10 min at 4 °C. The resultant supernatant was filtered through two layers of gauze, and the filtrate was centrifuged at 100,000 *g* for 30 min. The remaining pellet was suspended in extraction buffer containing protease inhibitors and incubated for 30 min at 4 °C under continuous stirring in the presence of 0.05% digitonin. Finally, the homogenate was centrifuged at 5,000 *g* for 15 min. The protein concentration in the supernatant was determined using the Bradford assay^68^.

For various assays based on centrifugation methods, the extracts from the 24 DPA cotton fiber powders were centrifuged at 2,000 *g* and filtered. The filtrates were centrifuged using two different methods: (1) centrifugation at 100,000 *g* for 30 min, after which the pellet was suspended in extraction buffer containing protease inhibitors and incubated for 30 min under continuous stirring in the presence of a final concentration of 0.05% digitonin; or (2) layering over a cushion of 60% sucrose and centrifugation at 100,000 *g* for 30 min, after which the membranes concentrated at the buffer-sucrose interface were incubated for 30 min under continuous stirring in the presence of a final concentration of 0.05% digitonin. To isolate the detergent-soluble membrane proteins, the homogenate was centrifuged at different speeds (5,000 *g*, 50,000 *g*, 100,000 *g*) for 15 min.

To investigate the role of cellulase in the extraction of membrane proteins, cellulase (C2605, Sigma, cellulase activity: ≥1,000 units g^−1^) was added to the extraction buffer at a final concentration of 0.25%, 0.50% or 1.0% (v/v), and the extracts were incubated at 37 °C for 10 min.

### Synthesis *in vitro* of β-1,4-glucan and β-1,3-glucan

Glucan synthesis reactions were performed *in vitro* as described by Peng *et al*.^38^. The reaction contained 120 μl of buffer [2.0 mM UDP-glucose, 2.0 μl 0.025 μCi μl^−1 14^C-UDP-glucose (11.174 GBq mmol^−1^; Perkin Elmer), 10 mM MgCl_2_, 2.0 mM CaCl_2_, 10 mM cellobiose] and 380 μl of a fresh digitonin membrane protein extract. The final 500 μl reaction solution was incubated at 25 °C for 2 h, then at 37 °C for an additional 2 h. The reaction was terminated by heating at 100 °C for 10 min. Each assay was independently repeated 3 times from the collected fiber tissues, and the molar ratio between the total UDP-glucose and 14C-labeled UDP-glucose was 7551:1.

The synthesized products of β-1,4-glucan (cellulose) and β-1,3-glucan (callose) were detected and subjected to the following sequential steps: (1) After termination of the reaction, ethanol was added to a final concentration at 70% (v/v), then mixed well and centrifuged at 18,000 *g* for 10 min at room temperature. The remaining pellet was washed several times with 70% ethanol until the radioactive reading for the supernatant obtained using a 1450 LSC & Luminescence counter was less than 50 cpm (Perkin Elmer, Microbeta Trilux). (2) The remaining pellet was washed with 0.7 ml of chloroform/methanol (1/1, v/v) for 1 h at 40 °C, followed by 0.7 ml of methanol for 30 min and 0.7 ml of acetone for 5 min. (3) The air-dried pellet was suspended in 500 μl of 0.1 M acetate buffer (pH 4.5) and incubated with 2.0 μl of 20 U ml^−1^ endo-β-1,3-glucanase from *Trichoderma sp.* (Megazyme E-LAMSE) and 0.4 μl of 100 U ml^−1^ exo-β-1,3-glucanase from *Trichoderma sp.* (Megazyme E-EXBGL) for 24 h at 37 °C. Enzymatic digestion was stopped by heating at 100 °C for 10 min, and the pellet was washed with 70% ethanol until the radioactive reading of the finally collected supernatant was below 50 cpm. All of the collected supernatants were combined and evaporated to a volume of 1.0 ml at 98 °C, and the resultant radioactivity was measured in 3.0 ml of a liquid scintillation mixture to quantify the callose product. (4) The final pellet was suspended in 500 μl of 0.12 M acetate buffer (pH 4.8), followed by incubation with 200 μl of 0.32 mg ml^−1^ cellulase complex (Imperial Jade Bio-technology) for 24 h at 37 °C. The reaction was stopped by heating at 100 °C for 10 min. After centrifugation, the pellet was washed with distilled water several times until the radioactive reading of the finally collected supernatant was below 50 cpm. All supernatants were combined, and the radioactivity was measured in 3.0 ml of liquid scintillation mixture to quantify the cellulose product. All experiments were conducted in biological triplicate.

### Immunoprecipitation and Western blot analysis

The antibody preparation was described previously by Li *et al*.^2^. The membrane pellet was suspended in extraction buffer containing protease inhibitors, followed by incubation for 30 min at 4 °C under continuous stirring in the presence of 2% Triton X-100. Finally, the homogenate was centrifuged at 5,000 *g* for 15 min. The protein concentration in the supernatant was determined using the Bradford assay^64^. The 500 μl supernatant (2% TrixonX-100 soluble) was mixed with 5 μl (9 μg) of anti-GhCESA1/8 or the pre-immune serum of anti-GhCESA8 (as control of protein identification by LC-MS/MS) and incubated for 1 h at 4 °C. Then, 40 μl of protein A-agarose was added to the mixture, which was gently shaken for 1 h at 4 °C with end-over-end rotation. After centrifugation for 1 min at 2,000 *g*, the harvested pellet was washed three times with ice-cold extraction buffer. The pellet was heated in 50 μl of sampling buffer at 70 °C for 5 min, then at 100 °C for 5 min. The obtained proteins were loaded into a 10% SDS-PAGE gel. Following electrophoresis separation, the proteins were transferred to a nitrocellulose membrane (Pall) and subjected to immunoblot analysis with GhCESA1- and GhCESA8-specific antibodies, which were detected using a horseradish peroxidase (HRP)-conjugated secondary antibody. Futhermore, the membranes were stained with 3, 3′-diaminobenzidine (DAB)/NiCl. The relative protein level was calculated using Quantity One software, and the experiments were performed in biological triplicate.

### Protein digestion and nanoflow liquid chromatography-tandem mass spectrometry (LC-MS/MS) analysis

The immunoprecipitated proteins were denatured and separated via SDS-PAGE. After staining with Coomassie Blue R-250, the protein bands (except the antibody bands) were cut from the gel together. The protein bands were then destained with 100 μl of a solution containing 50% v/v acetonitrile (ACN) and 25 mM ammonium bicarbonate for 1 h with three repeats. The gel blocks were dehydrated with 100 μl of 100% ACN for 40 min and dried in a SpeedVac concentrator for 30 min. The gels were then incubated with a 10 ng μl^−1^ trypsin solution in 25 mM ammonium bicarbonate at 37 °C for 12 h for hydrolysis. The hydrolyzed peptide mixtures were extracted twice with 8 μl of 50% v/v ACN and 0.5% v/v formic acid (FA). The extracts were dried under the protection of N_2_ and re-suspended with 5% ACN in 0.1% FA for LC-MS/MS analysis.

The nano-LC MS/MS experiment was performed in an HPLC system composed of two LC-20AD nano-flow LC pumps, an SIL-20 AC auto-sampler and an LC-20AB microflow LC pump (all Shimadzu, Tokyo, Japan) connected to an LTQ-Orbitrap mass spectrometer (ThermoFisher, San Jose, CA). The sample was loaded onto a CAPTRAP column (0.5 × 2 mm, MICHROM Bioresources, Auburn, CA) over 3 min at a flow rate of 50 μl min^−1^. The sample was subsequently separated with a C18 reverse-phase column (0.1 × 150 mm, packed with 3 μm Magic C18-AQ particles, MICHROM Bioresources, Auburn CA) at a flow rate of 500 nl min^−1^. The mobile phases were 5% acetonitrile with 0.1% formic acid (phase A and the loading phase) and 95% acetonitrile with 0.1% formic acid (phase B). To achieve proper separation, a 90-min linear gradient from 5 to 45% phase B was employed. The separated sample was introduced into the mass spectrometer via an ADVANCE 30 μm silica tip (MICHROM Bioresources, Auburn CA). The spray voltage was set at 2.3 kV, and the heated capillary remained at 200 °C. The mass spectrometer was operated in data-dependent mode, and each duty cycle consisted one full-MS survey scan over the mass range of 400~2,000 Da, with resolution power of 100,000 using the Orbitrap section, followed by MS/MS analyses of the 10 strongest peaks using the LTQ section. The AGC expectations during full-MS and MS/MS were 1000,000 and 10,000, respectively. The peptides were fragmented in the LTQ section using collision-induced dissociation with helium, with the normalized collision energy value set at 35%. Only 2+ and 3+ peaks were selected for MS/MS runs, and previously fragmented peptides were excluded for 60 s.

Protein searches were performed against the NCBI cotton protein database (release data: 20130425; 3,559 protein sequences) using Mascot 2.3.02 software (www. matrixscience.com) with the following criteria: 2 possible missed cleavage sites; the enzyme set to trypsin; a peptide mass tolerance of 20 ppm; a fragment mass tolerance of 0.80 Da; and an acetylated protein N-term and oxidized Met were considered possible modifications. The acceptance criterion for peptide identification was a rate of false positive identification below 1% and percolater score greater than 13. Levels of the identified proteins were relatively measured by calculating total peptide counts and distinct peptide numbers^48^,^49^. All experiments were performed in biological triplicate.

### Real-time PCR

Total RNA was isolated from the collected tissues using the method described by Wu and Liu^69^, with minor modifications as indicated by Li *et al*.^2^. First-strand cDNA was obtained using M-MLV reverse transcriptase (Promega). Real-time PCR amplification was carried out on a Bio-Rad MyCycler thermal cycler with SYBER Premix ExTaq (TakaRa) according to the manufacturer’s instruction, and *GhUBQ7* was used as the internal control. The expression value of *GhUBQ7* was defined as 100, and the expression level of nine GhCESA genes were thus normalized with the expression level of *GhUBQ7.* All of the primers used in these assays are listed in Supplementary Table S8, and the assays were carried out in biological triplicates.

### Co-expression analysis of *Arabidopsis* genes

Microarray data from *Arabidopsis* were downloaded from the Gene Expression Omnibus database (GEO, http://www.ncbi.nlm.nih.gov/geo/) using GSE series accession numbers GSE5629, GSE5630, GSE5631, GSE5632, GSE5633 and GSE5634. The raw data were processed with the Affymetrix Microarray Analysis Suite (MAS Version 5, Affymetrix). Genes that could not be found in the CEO database were not further investigated in this work. Subsequent analysis of the gene expression data was performed in the statistical computing language R (http://www.rproject.org) using packages available from the Bioconductor project (http://www.bioconductor.org, 48).

### Statistical calculation of correlation coefficients

Correlation coefficients were calculated by performing Spearman rank correlation analysis for all pairs of the tested genes. The obtained correlations were subjected to significance tests at *P* < 0.05, and non-correlation was defined as *P* > 0.05.

## Additional Information

**How to cite this article**: Li, A. *et al*. Proteomic profiling of cellulase-aid-extracted membrane proteins for functional identification of cellulose synthase complexes and their potential associated-components in cotton fibers. *Sci. Rep.* **6**, 26356; doi: 10.1038/srep26356 (2016).

## Supplementary Material

Supplementary Information

Supplementary Information

## Figures and Tables

**Table 1:** β-1,4-glucan and β-1,3-glucan products synthesized *in vitro* using different ultracentrifugation methods for the extraction of membrane proteins from cotton fibers.

**Ultracentrifugation method**	**Glucan**	**Counts per minute (cpm) incorporated (cpm mg**^**−1**^ **protein)**	**SE/SU**	**Total nmol of glucose incorporated (nmol mg**^**−1**^ **protein)**	**SE/SU**
Sucrose density (SU)	β-1,4-glucan	5,670 ± 190		50.0 ± 2.0	
	β-1,3-glucan	12,900 ± 1,000		113 ± 9.0	
Sedimentation velocity (SE)	β-1,4-glucan	10,100 ± 400	1.78	89.0 ± 3.0	1.78
	β-1,3-glucan	22,200 ± 1,500	1.72	195 ± 14	1.73

The data presented as mean ± SD (n = 3).

**Table 2:** Detectable peptide numbers of cellulose synthases (CESA) and callose synthase (CALS) in cotton fibers detected through LC-MS/MS analysis.

**Protein name**	**Total peptides (Exp 1)**			**Total peptides (Exp 2)**			**Total peptides (Exp 3)**			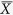 *
	**Blank (B)**	**Treated (T)**		**Blank (B)**	**Treated (T)**		**Blank (B)**	**Treated (T)**		
GhCESA1	49(23)@	82(34)	**67%(48%)**#	59(23)	97(26)	**64%(13%)**	42(25)	58(27)	**38%(8%)**	**56% (23%)**
GhCESA2	48(24)	82(32)	**71%(33%)**	31(17)	66(20)	**112%(18%)**	22(16)	32(19)	**45%(19%)**	**76%(23%)**
GhCESA7	40(20)	55(23)	**38%(15%)**	46(15)	70(21)	**52%(40%)**	35(17)	55(24)	**57%(41%)**	**49%(32%)**
GhCESA8	50(22)	69(27)	**38%(23%)**	63(18)	98(22)	**56%(22%)**	42(17)	62(25)	**48%(47%)**	**47%(31%)**
GhCALS	3(3)	4(3)	**33%(0)**	1(1)	4(3)	**300%(200%)**	1(1)	11(8)	**1000%(700%)**	**444%(300%)**

(B)/(T): Treated without/with 1% cellulose.

^@^Indicated total peptide counts and distinct peptide numbers (brackets).

^#^Indicated ratio (%) of the increased total peptide counts and distinct peptide numbers (brackets) upon cellulase treatment by calculating (T)-(B)/(B).

^*^Indicated average of the increased total peptide counts and distinct peptide numbers in the three independent experiments (Exp).

**Table 3:** Identification of the five well-known proteins associated with cellulose synthase complex in cotton fibers by LC-MS/MS analysis.

**Protein name**	**Total peptides (Exp 1)**		**Total peptides (Exp 2)**		**Total peptides (Exp 3)**		**Accession number**	**TM domain**	**Protein fuction**
	**Blank (B)**	**Treated (T)**	**Blank (B)**	**Treated (T)**	**Blank (B)**	**Treated (T)**			
alpha-tubulin 4	20(10)@	22(10)	14(6)	14(7)	0(0)	12(9)	gi|37529490	0	Cytoskeleton, GTPase activity, guidance of cellulose deposition^25^,^26^,^30^
beta-tubulin 3	25(10)	23(10)	30(10)	19(8)	33(11)	0(0)	gi|223453022	0	Cytoskeleton, GTPase activity, protein binding, guidance of cellulose deposition^25^,^26^,^30^
beta-tubulin 7	19(7)	23(8)	33(12)	20(8)	37(11)	32(12)	gi|37529498	0	Cytoskeleton, GTPase activity, protein binding, guidance of cellulose deposition^25^,^26^,^30^
endo-1,4-beta-glucanase (KOR)	3(2)	2(2)	21(8)	14(5)	23(12)	25(13)	gi|32454474	1	cellulase activity, cellulose biosynthesis^21^,^22^,^24^,^32^,^33^,^38^,^57^
sucrose synthase 1	6(3)	4(2)	10(6)	9(5)	20(13)	16(12)	gi|258489633	0	UDP-glycosyltransferase activity, cellulose biosynthesis^20^,^23^,^31^

(B)/(T): Treated without/with 1% cellulose.

^@^Indicated total peptide counts and distinct peptide numbers (brackets); TM: Transmembrane.

**Table 4:** Classification of the novel proteins associated with cellulose and callose synthase complexes in cotton fibers.

**Group**	Protein name*	**Total peptides (Exp 1)**		**Total peptides (Exp 2)**		**Total peptides (Exp 3)**		**Accession number**	**TM domain**	**Protein fuction**
		**Blank (B)**	**Treated (T)**	**Blank (B)**	**Treated (T)**	**Blank (B)**	**Treated (T)**			
CESA-associated										
I	Phenylcoumaran benzylic ether reductase-like protein	3(2)@	8(5)	2(1)	3(2)	3(3)	6(5)	gi|124488476	0	isoflavone reductase
	vacuolar H^+^ -ATPase catalytic subunit	8(4)	16(9)	0(0)	4(3)	11(9)	15(8)	gi|167313	0	cytoskeleton, ATP binding, hydrolase activity
II	glyceraldehyde-3-phosphate dehydrogenase C subunit	48(18)	44(18)	37(12)	17(8)	16(12)	16(12)	gi|211906518	0	NAD binding, NADP binding, oxidoreductase activity
	fasciclin-like arabinogalactan protein	2(1)	2(2)	2(1)	7(4)	5(4)	5(5)	gi|606942	1	cell wall biogenesis, acetyl-CoA metabolic process
	UDP-glucuronic acid decarboxylase 2	15(7)	20(8)	8(5)	0(0)	10(6)	21(11)	gi|213950353	1	UDP-xylose metabolic, UDP-glucuronate decarboxylase activity
	tonoplast intrinsic protein	0(0)	1(1)	2(1)	2(1)	1(1)	1(1)	gi|227434194	6	water channel, facilitate the transport of urea and hydrogen peroxide
CALS-associated										
I	ubiquitin extension protein	8(5)	12(5)	5(3)	7(3)	9(4)	10(4)	gi|73761683	0	structural constituent of ribosome, ubiquitin-dependent protein catabolic process
	3-ketoacyl-CoA reductase 1	5(3)	19(10)	1(1)	5(3)	6(3)	8(5)	gi|62956018	1	acetoacetyl-CoA reductase, ketoreductase, oxidoreductase
II	plasma membrane H+ -ATPase, partial	2(1)	3(2)	0(0)	2(1)	2(2)	2(2)	gi|2911803	9	ATPase activity, cation transport

TM: Transmembrane; I: Proteins showing more total peptide counts and distinct peptide numbers upon cellulase treatment; II: Proteins without more detectable peptides upon cellulase treatment.

^*^Based on bioinformatic analysis of *Arabidopsis* homologs of the 19 cotton fiber proteins identified via LC-MS/MS (Supplementary Table S7); (B)/(T): Treated without/with 1% cellulose.

^@^Indicated total peptide counts and distinct peptide numbers (brackets).

**Table 5:** Comparison of anti-CESA-immunoprecipitated proteins between cotton fibers and *Populus* xylem fibers.

**No**	**Cotton protein name**	**Cotton protein ID (NCBI database)**	***Poplus*** **protein name**	**Poplus Protein ID (JGI Database)**
1	CESA1	gi|324984035	CesA8-A/B	235238/555650
2	CESA2	gi|219907965	CesA4	553321
3–4	CESA7/8	gi|376315425/gi|376315428	CesA7-A/B	717644/262611
5	KOR	gi|345103975	KOR	675657
6	sucrose synthase 1	gi|258489633	sucrose synthase	835735/826368
7–9	alpha-tubulin/beta-tubulin	gi|37529490/gi|37529498/gi|223453022	tubulin	641120
10	callose synthase	gi|4588012	callose synthase 1	814785
11	fasciclin-like arabinogalactan protein	gi|606942	fasciclin-like arabinogalactan protein 13	730906
12	UDP-glucuronic acid decarboxylase 2	gi|213950353	UDP-glucuronic acid decarboxylase 1	735401
