# Supplementary material for: Proteomic profiling of cellulase-aid-extracted membrane proteins for functional identification of cellulose synthase complexes and their potential associated- components in cotton fibers
Source: Sci Rep. 2016 May 19;6:26356. doi: 10.1038/srep26356 (PMC4872218; doi:10.1038/srep26356)
Supplement: Supplementary Information [file srep26356-s1.pdf]

Proteomic profiling of cellulase-aid-extracted membrane proteins for functional identification of cellulose synthase complexes and their potential associated-components in cotton fibers

Ao Li[1,2,3], Ruyi Wang[1,2,3], Xianliang Li[1,2,3], Mingyong Liu[1,2,4], Jian Fan[1,2,3], Kai Guo[1,2,4], Bing Luo[5], Tingting Chen[1,2,4], Shengqiu Feng[1,2,3], Yanting Wang[1,2,3], Bingrui Wang[1,2,3], Liangcai Peng[1,2,3], Tao Xia[1,2,4*]

Supplementary Information

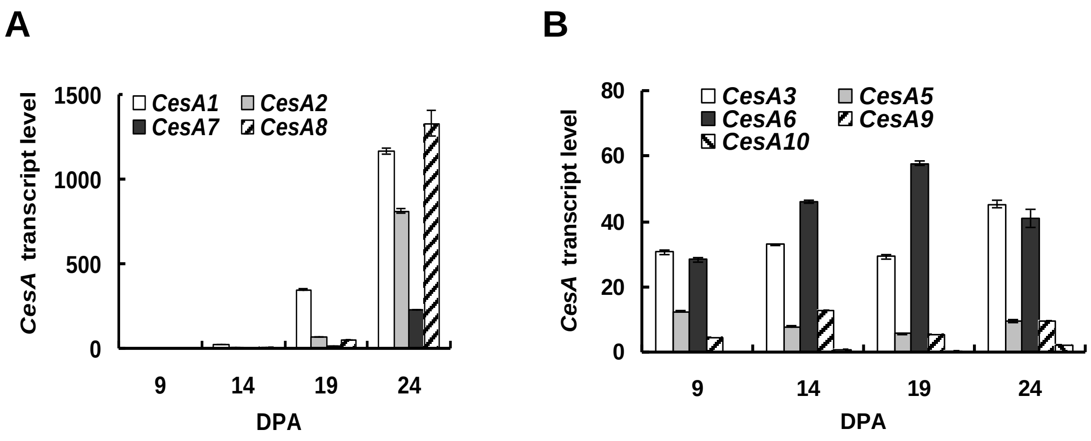

Figure S1. Transcript levels of nine *GhCesA* genes during the development of cotton fibers. Error bars indicate SD (standard deviation) (n=3).

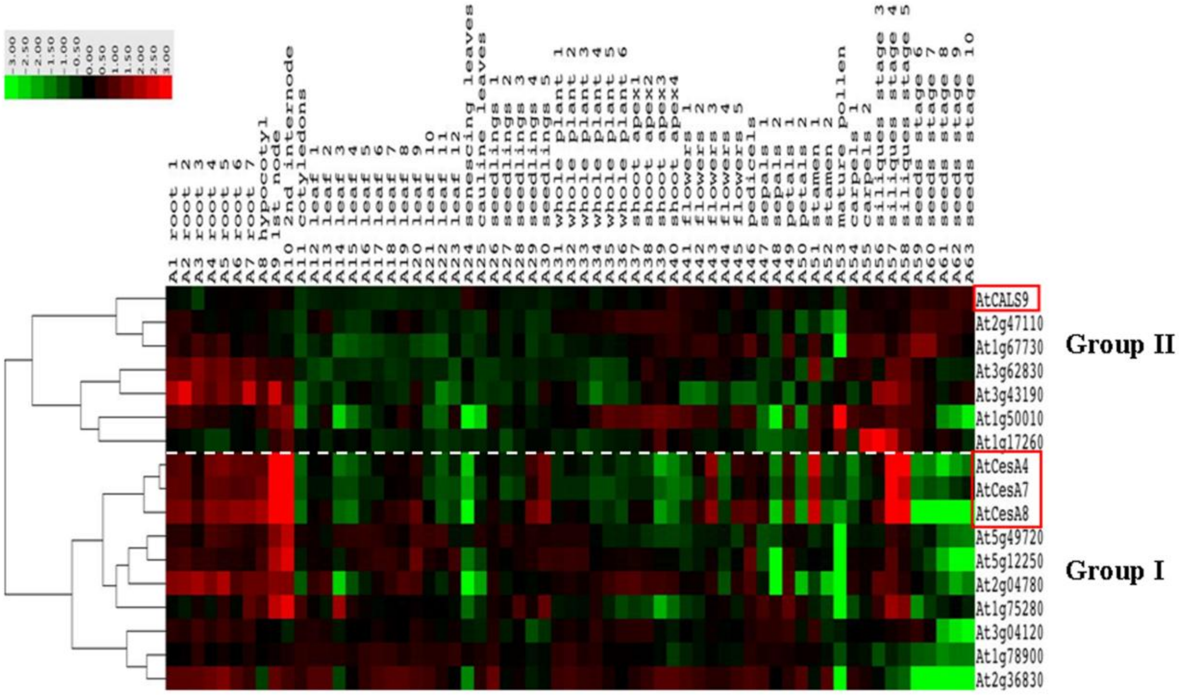

Figure S2. Gene expression profiling of *Arabidopsis* genes encoding homologs of anti-GhCESA8-precipitated proteins from cotton fibers.

Table S1. β-1,4-Glucan and β-1,3-glucan products synthesized *in vitro* from total membrane extracts of the cotton fibers treated with/without cellulase.

Cellulase added (%)	β-1,4-glucan			β-1,3-glucan		
	Counts per minute (cpm) incorporated (cpm mg^{-1} protein)	Glucose incorporated (nmol mg^{-1} protein)	Ratio(cellulase /no cellulase)	Cpm incorporated (cpm mg^{-1} protein)	Glucose incorporated (nmol mg^{-1} protein)	Ratio(cellulase/ no cellulase)
0	6,720 ± 810	62.0 ± 7.0	1	21,300 ± 1,300	187 ± 11	1
0.25%	9,790 ± 90	91.0 ± 1.0	1.5	48,900 ± 200	430 ± 2	2.3
0.50%	20,900 ± 300	190 ± 3	3.1	70,500 ± 3,100	620 ± 27	3.4
1.00%	24,700 ± 800	223 ± 7	3.6	75,300 ± 2,400	662 ± 21	3.5

The data are presented as the mean ± SD (*n*=3).

Table S2. The cellulose synthase (CESA) family identified in *Gossypium* and *Arabidopsis thaliana*.

Homologous genes in *G. raimondii*[1] (Gene name)	Homologous genes in *G. hirsutum* TM-1[2] (Gene name)	Homologous genes in *G. hirsutum* Huamian 99 (Gene name)	Homologous genes in *Arabidopsis thaliana* (Gene name)
CESA1 Clade			
GrCESA1-A	GhCESA1A_A05	GhCESA6	AtCESA1
	GhCESA1A_D05		AtCESA10
GrCESA1-B	GhCESA1B_A05		
	GhCESA1B_D05		
CESA3 Clade			
GrCESA3-A	GhCESA3A_A08	GhCESA3	AtCESA3
	GhCESA3A_D08	GhCESA10	
GrCESA3-B	GhCESA3B_A08		
	GhCESA3B_D08		
GrCESA3-C	GhCESA3C_A02		
	GhCESA3C_D03		
CESA6 Clade			
GrCESA6-A	GhCESA6A_A02	GhCESA5	AtCESA2
	GhCESA6A_D03	GhCESA9	AtCESA5
GrCESA6-B	GhCESA6B_A05		AtCESA6
	GhCESA6B_D05		AtCESA9
GrCESA6-C	GhCESA6C_A06		
	GhCESA6C_D06		
	GhCESA6_D12		
GrCESA6-D	GhCESA6D_A11		
	GhCESA6D_D11		
	GhCESA6_A13		
	GhCESA6_D13		
CESA4 Clade			
GrCESA4-A	GhCESA4A_A07	GhCESA2	AtCESA4
	GhCESA4A_D07		
GrCESA4-B	GhCESA4B_A08		
	GhCESA4B_D08		
CESA7 Clade			
GrCESA7-A	GhCESA7A_A07	GhCESA7	AtCESA7
	GhCESA7A_D07	GhCESA8	

GrCESA7-B	GhCESA7B_A05		
	GhCESA7B_D05		
CESA8 Clade			
GrCESA8-A	GhCESA8A_D05	GhCESA1	AtCESA8
GrCESA8-B	GhCESA8B_A10		
	GhCESA8B_D10		

1 Paterson, A. H. et al. Repeated polyploidization of *Gossypium* genomes and the evolution of spinnable cotton fibres. *Nature* ***492,*** 423-428 (2012).

2 Zhang, T. Z. et al. Sequencing of allotetraploid cotton (*Gossypium hirsutum* l. acc. TM-1) provides a resource for fiber improvement. *Nat. Biotechnol.* **33(5),** 531-537 (2015).

Table S3. Specific detectable peptide numbers of four GhCESA proteins in cotton fibers by LC-MS/MS analysis.

Protein	Specific peptides (Exp 1)			Specific peptides (Exp 2)			Specific peptides (Exp 3)			Means*
	Blank (B)	Treated (T)		Blank (B)	Treated (T)		Blank (B)	Treated (T)		
GhCESA1	16	25	56%#	15	20	33%	20	22	10%	33%
GhCESA2	15	21	40%	9	14	56%	10	12	20%	39%
GhCESA7	2	3	50%	4	8	100%	4	5	25%	58%
GhCESA8	5	7	40%	7	9	29%	5	6	20%	30%

(B)/(T): Treated without/with 1% cellulase; #Increased peptide number ratio (%) upon cellulase treatment: (T)-(B)/(B); *Average of the increased peptide number ratios from three independent experiments (Exp).

Table S4. Total 32 proteins detected by LC-MS/MS from the anti-GhCESA8 immunoprecipitation and the pre-immune serum in the cotton fibers.

Group	Protein name	Accession number	Pulled down by		
			Anti-GhCESA8 (A)	Pre-immune serum (P)	
	GhCESA1	gi|324984035	52(22)[#]	13(10)	300%(120%)[*]
	GhCESA2	gi|219907965	49(18)	11(8)	345%(125%)
	GhCESA7	gi|376315426	41(17)	9(6)	356%(183%)
	GhCESA8	gi|376315428	46(18)	9(6)	411%(200%)
I	Endo-1,4-beta-glucanase (KOR)	gi|32454474	17(9)	5(3)	240%(200%)
	ubiquitin extension protein	gi|73761683	4(2)	1(1)	300%(100%)
	fasciclin-like arabinogalactan protein	gi|606942	4(3)	1(1)	300%(200%)
	UDP-glucuronic acid decarboxylase 2	gi|213950353	4(4)	1(1)	300%(300%)
	beta-tubulin 3	gi|223453022	14(8)	0(0)	-
	beta-tubulin 7	gi|37529498	12(7)	0(0)	-
	Alpha-tubulin 4	gi|37529490	13(6)	0(0)	-
	sucrose synthase 1	gi|258489633	3(3)	0(0)	-
	GhCALS	gi|4588012	2(2)	0(0)	-
II	glyceraldehyde-3-phosphate dehydrogenase C subunit	gi|211906518	5(5)	0(0)	-
	3-ketoacyl-CoA reductase 1	gi|62956018	4(4)	0(0)	-
	plasma membrane H^+-ATPase, partial	gi|2911803	1(1)	0(0)	-
	vacuolar H^+-ATPase catalytic subunit	gi|167313	2(1)	0(0)	-
	tonoplast intrinsic protein	gi|227434194	1(1)	0(0)	-
	phenylcoumaran benzylic ether reductase-like protein	gi|124488476	1(1)	0(0)	-
	tubulin beta-1	gi|19569135	15(8)	7(4)	114%(100%)
	adenine nucleotide translocator 1	gi|2463664	10(8)	8(7)	25%(14%)
	luminal binding protein	gi|211906506	7(5)	3(2)	133%(150%)
	pyrophosphate-energized vacuolar membrane proton pump	gi|307948772	4(3)	3(2)	33%(50%)
	Annexin	gi|211906452	4(3)	2(1)	100%(200%)
III	translation elongation factor 1A-1	gi|74486728	2(1)	1(1)	100%(0%)
	UDP-glucuronic acid decarboxylase 1	gi|209420680	2(2)	1(1)	100%(100%)
	CC-NBS-LRR protein	gi|110725269	2(1)	1(1)	100%(0%)
	fasciclin-like arabinogalactan protein	gi|30841338	5(3)	3(3)	67%(0%)
	Actin	gi|32186890	11(6)	11(6)	0%(0%)
	pyruvate dehydrogenase alpha subunit	gi|211906500	3(3)	5(5)	-40%(-25%)
	adenylate kinase	gi|295422151	1(1)	2(1)	-50%(0%)
	ribosomal protein, small subunit 4e	gi|488739	4(4)	4(3)	0%(33%)

#Indicated total peptide counts and distinct peptide numbers (brackets), *Indicated ratio (%) of the increased total peptide counts and distinct peptide numbers (brackets) using anti-GhCESA8 and pre-immune serum by calculating (A)-(P)/(P); Group I: proteins showing total peptide counts in precipitation using anti-GhCESA8 by 2-4 folds more than that of the pre-immune serum; Group II: proteins specifically detected in precipitation using anti-GhCESA8, other than the pre-immune serum; Group III: proteins showing total peptide counts in precipitation using anti-GhCESA8 by less than 2-folds compared with the pre-immune serum.

Table S5. Total 19 proteins detected by LC-MS/MS from the anti-GhCESA8 immunoprecipitation in the cotton fibers treated with 1% cellulase in three independent experiments.

Group@	Protein name	Total peptides (Exp 1)*		Total peptides (Exp 2)		Total peptides (Exp 3)		Accession number
		Blank (B)	Treated (T)	Blank (B)	Treated (T)	Blank (B)	Treated (T)	
I	GhCESA1	49(23)#	82(34)	59(23)	97(26)	42(25)	58(27)	gi|324984035
	GhCESA2	48(24)	82(32)	31(17)	66(20)	22(16)	32(19)	gi|219907965
	GhCESA7	40(20)	55(23)	46(15)	70(21)	35(17)	55(24)	gi|376315426
	GhCESA8	50(22)	69(27)	63(18)	98(22)	42(17)	62(25)	gi|376315428
	alpha-tubulin 4	20(10)	22(10)	14(6)	14(7)	0(0)	12(9)	gi|37529490
	beta-tubulin 3	25(10)	23(10)	30(10)	19(8)	33(11)	0(0)	gi|223453022
	beta-tubulin 7	19(7)	23(8)	33(12)	20(8)	37(11)	32(12)	gi|37529498
	KOR	3(2)	2(2)	21(8)	14(5)	23(12)	25(13)	gi|32454474
	Sucrose synthase 1	6(3)	4(2)	10(6)	9(5)	20(13)	16(12)	gi|258489633
II	GhCALS	3(3)	4(3)	1(1)	4(3)	1(1)	11(8)	gi|4588012
III	glyceraldehyde-3-phosphate dehydrogenase C subunit	48(18)	44(18)	37(12)	17(8)	16(12)	16(12)	gi|211906518
	UDP-glucuronic acid decarboxylase 2	15(7)	20(8)	8(5)	0(0)	10(6)	21(11)	gi|213950353
	vacuolar H^+-ATPase catalytic subunit	8(4)	16(9)	0(0)	4(3)	11(9)	15(8)	gi|167313
	ubiquitin extension protein	8(5)	12(5)	5(3)	7(3)	9(4)	10(4)	gi|73761683
	3-ketoacyl-CoA reductase 1	5(3)	19(10)	1(1)	5(3)	6(3)	8(5)	gi|62956018
	plasma membrane H^+-ATPase, partial	2(1)	3(2)	0(0)	2(1)	2(2)	2(2)	gi|2911803
	fasciclin-like arabinogalactan protein	2(1)	2(2)	2(1)	7(4)	5(4)	5(5)	gi|606942
	phenylcoumaran benzylic ether reductase-like protein	3(2)	8(5)	2(1)	3(2)	3(3)	6(5)	gi|124488476
	tonoplast intrinsic protein	0(0)	1(1)	2(1)	2(1)	1(1)	1(1)	gi|227434194

*(B)/(T): Treated without/with 1% cellulase; @Group I: Cellulose synthase complex and five well-known cellulose synthase complex associated-proteins; Group II: Callose synthase; Group III: Novel proteins; # total peptide counts and distinct peptide numbers (brackets).

Table S6. Tissue samples obtained from different developmental stages in *Arabidopsis*.

Sample No	Abbreviation	Tissues/organ and developmental stages
A1	root 1	root roots Wt 7 days
A2	root 2	root roots Wt 17 days
A3	root 3	root root Wt 15 days
A4	root 4	root root Wt 8 days
A5	root 5	root root Wt 8 days
A6	root 6	root root Wt 21 days
A7	root 7	root root Wt 21 days
A8	hypocotyl	stem hypocotyl Wt 7 days
A9	1st node	stem 1st node Wt 21+ days
A10	2nd internode	stem stem, 2nd internode Wt 21+ days
A11	cotyledons	leaf cotyledons Wt 7 days
A12	leaf 1	leaf leaves 1 + 2 Wt 7 days
A13	leaf 2	leaf rosette leaf #4, 1 cm long Wt 10 days
A14	leaf 3	leaf rosette leaf # 2 Wt 17 days
A15	leaf 4	leaf rosette leaf # 4 Wt 17 days
A16	leaf 5	leaf rosette leaf # 6 Wt 17 days
A17	leaf 6	leaf rosette leaf # 8 Wt 17 days
A18	leaf 7	leaf rosette leaf # 10 Wt 17 days
A19	leaf 8	leaf rosette leaf # 12 Wt 17 days
A20	leaf 9	leaf leaf 7, petiole Wt 17 days
A21	leaf 10	leaf leaf 7, proximal half Wt 17 days
A22	leaf 11	leaf leaf 7, distal half Wt 17 days
A23	leaf 12	leaf leaf Wt 15 days
A24	senescing leaves	leaf senescing leaves Wt 35 days
A25	cauline leaves	leaf cauline leaves Wt 21+ days
A26	seedlings 1	whole plant seedling, green parts Wt 7 days
A27	seedlings 2	whole plant seedling, green parts Wt 8 days
A28	seedlings 3	whole plant seedling, green parts Wt 8 days
A29	seedlings 4	whole plant seedling, green parts Wt 21 days
A30	seedlings 5	whole plant seedling, green parts Wt 21 days
A31	whole plant 1	whole plant developmental drift, entire rosette after transition to flowering, but before bolting Wt 21 days
A32	whole plant 2	whole plant as above Wt 22 days
A33	whole plant 3	whole plant as above Wt 23 days
A34	whole plant 4	whole plant vegetative rosette Wt 7 days
A35	whole plant 5	whole plant vegetative rosette Wt 14 days
A36	whole plant 6	whole plant vegetative rosette Wt 21 days
A37	shoot apex1	apex shoot apex, vegetative + young leaves Wt 7 days
A38	shoot apex2	apex shoot apex, vegetative Wt 7 days
A39	shoot apex3	apex shoot apex, transition (before bolting) Wt 14 days
A40	shoot apex4	apex shoot apex, inflorescence (after bolting) Wt 21 days

A41	flowers 1	flowers flowers stage 9 Wt 21+ days
A42	flowers 2	flowers flowers stage 10/11 Wt 21+ days
A43	flowers 3	flowers flowers stage 12 Wt 21+ days
A44	flowers 4	flowers flowers stage 15 Wt 21+ days
A45	flowers 5	flowers flower Wt 28 days
A46	pedicels	flowers stage 15, pedicels Wt 21+ days
A47	sepals 1	flowers stage 12, sepals Wt 21+ days
A48	sepals 2	flowers stage 15, sepals Wt 21+ days
A49	petals 1	flowers stage 12, petals Wt 21+ days
A50	petals 2	flowers stage 15, petals Wt 21+ days
A51	stamen 1	flowers stage 12, stamens Wt 21+ days
A52	stamen 2	flowers stage 15, stamen Wt 21+ days
A53	mature pollen	mature pollen Wt 6 wk
A54	carpels 1	flowers stage 12, carpels Wt 21+ days
A55	carpels 2	flowers stage 15, carpels Wt 21+ days
A56	siliques stage 3	seeds siliques, w/ seeds stage 3; mid globular to early heart embryos Wt 8 wk
A57	siliques stage 4	seeds siliques, w/ seeds stage 4; early to late heart embryos Wt 8 wk
A58	siliques stage 5	seeds siliques, w/ seeds stage 5; late heart to mid torpedo embryos Wt 8 wk
A59	seeds stage 6	seeds seeds, stage 6, w/o siliques; mid to late torpedo embryos Wt 8 wk
A60	seeds stage 7	seeds seeds, stage 7, w/o siliques; late torpedo to early walking-stick embryos Wt 8 wk
A61	seeds stage 8	seeds seeds, stage 8, w/o siliques; walking-stick to early curled cotyledons embryos Wt 8 wk
A62	seeds stage 9	seeds seeds, stage 9, w/o siliques; curled cotyledons to early green cotyledons embryos Wt 8 wk
A63	seeds stage 10	seeds seeds, stage 10, w/o siliques; green cotyledons embryos Wt 8 wk

Table S7. Coexpression analysis of *Arabidopsis* genes encoding homologs of anti-GhCESA8-precipitated proteins.

Group	Cotton protein name	Accession Number	*Arabidopsis* homolog gene	Secondary cell wall CesAs			Callose synthase gene
				AtCesA4	*AtCesA7*	*AtCesA8*	*AtCALS9*
I	alpha-tubulin 4	gi|37529490	At1g50010	.376**	.300*	.409**	0.167
	beta-tubulin 3	gi|223453022	At5g12250	.718**	.708**	.728**	-.328**
	beta-tubulin 7	gi|37529498	At5g12250	.718**	.708**	.728**	-.328**
	KOR	gi|32454474	At5g49720	.535**	.535**	.536**	-.292*
	sucrose synthase 1	gi|258489633	At3g43190	0.246	.312*	.277*	0.130
II	ubiquitin extension protein	gi|73761683	At2g47110	0.133	0.063	0.137	.407**
	3-ketoacyl-CoA reductase 1	gi|62956018	At1g67730	0.179	0.137	0.16	.550**
	plasma membrane H^+-ATPase, partial	gi|2911803	At1g17260	-0.122	-0.077	-0.058	.282*
	vacuolar H^+-ATPase catalytic subunit	gi|167313	At1g78900	.296*	.275*	0.235	-.369**
	phenylcoumaran benzylic ether reductase-like protein	gi|124488476	At1g75280	.485**	.499**	.486**	-.258*
III	glyceraldehyde-3-phosphate dehydrogenase C subunit	gi|211906518	At3g04120	.457**	.381**	.426**	0.082
	fasciclin-like arabinogalactan protein	gi|606942	At2g04780	.687**	.646**	.689**	-0.1
	UDP-glucuronic acid decarboxylase 2	gi|213950353	At3g62830	.403**	.436**	.521**	0.232
	tonoplast intrinsic protein	gi|227434194	At2g36830	.390**	.352**	.353**	-0.028

&*Indicate gene co-expression with significant correlations at $P < 0.05$ and 0.01, respectively, with (-) indicating a negative correlation, while the others are positive. Group I: five well-known proteins associated with cellulose synthase complex; Group II: proteins showing more detectable peptide numbers upon cellulase treatment; Group III, proteins not showing more detectable peptides upon cellulase treatment.

Table S8. Primers used for real-time PCR.

Primer Name	Sequences(5’-3’)	Accession number
Gh*CesA1*QRT-F	CCTCAACAAAGGGTACGAAGC	U58283
Gh*CesA1*QRT-R	CAACACTGACCAAAGGACAACA	
Gh*CesA2*QRT-F	GTAGACACCAACTTCACCGTAACA	U58284
Gh*CesA2*QRT-R	GCCACGACTCCGACCATGT	
Gh*CesA3*QRT-F	ACAGTTTTGGGTTATCGGTGG	AF150630
Gh*CesA3*QRT-R	ATGATGAGGAGAGTGGTTGGTG	
Gh*CesA5*QRT-F	ATGACTGGTGGAGGAATGAGC	JQ345693
Gh*CesA5*QRT-R	CCAATAACGTGGTAGGAGGGA	
Gh*CesA6*QRT-F	TTCTCATTCCTCCAACCACAGT	JQ345694
Gh*CesA6*QRT-R	TTGACGACCCACCAAACCT	
Gh*CesA7*QRT-F	CTAGCAGGGATCGACACTAATTT	JQ345695
Gh*CesA7*QRT-R	GAGAAGATTGAAGCCAGCAGAA	
Gh*CesA8*QRT-F	CCGGCATCTCAGATGCCATA	JQ345696
Gh*CesA8*QRT-R	TTGAAGCCAATAGCACTGACC	
Gh*CesA9*QRT-F	CGGTGGTGTCTCGTCTCAC	JQ345697
Gh*CesA9*QRT-R	CCGACTATGACTCCGATTATGT	
Gh*CesA10*QRT-F	ATGAGGTGGAGTGGGGTAGG	JQ345699
Gh*CesA10*QRT-R	AGTAGTTGGAGGAATGAGTAGGGTAG	
Gh*UBQ7*QRT-F	GAAGGCATTCCACCTGACCAAC	DQ116441
Gh*UBQ7*QRT-R	CTTGACCTTCTTCTTCTTGTGCTTG	
